# Reduction of hospital transfers: After integration of an advanced practice registered nurse practitioner and situation-background-appearance-review and notify form

**DOI:** 10.1016/j.ijnsa.2021.100041

**Published:** 2021-08-18

**Authors:** Robin D. Burgess

**Affiliations:** Capella University, 11750 Old Georgetown Road Unit 2410, Rockville, MD 20852, United States

**Keywords:** Hospital transfer, INTERACT program, Nursing home facility, Nurse practitioner

## Abstract

**Objectives:**

This article aims to address the hypothesis that the incorporation of the use of an Advanced Practice Registered Nurse and the Situation-Background-Appearance-Review and Notify form from the Interventions to Reduce Acute Care Transfers program will reduce the number of hospital transfers from nursing home facilities.

**Methods:**

This hypothesis was measured using de-identified data on hospital transfers from three facilities, as well as de-identified Situation-Background-Appearance-Review and Notify and Quality Initiative forms completed by the nursing staff at these facilities.

**Results:**

The collective number of hospital transfers seen over the eight-week pre-intervention period was 115 compared with the collective number of transfers seen over the eight-week post-intervention period which was 75. This represents an 34.8% overall reduction in hospital transfers.

**Conclusions:**

Implications of this project include the confirmation that the use of the Situation-Background-Appearance-Review and Notify tool integration of an Advanced Practice Registered Nurse can help reduce hospital transfers. These findings can help to initiate future projects and protocol changes.

**What is already known about the topic?**

• Advanced Registered Nurse Practitioners (APRN's) have been linked to reduced rates of hospital transfers when incorporated into nursing home facility environments.
• Hospital Transfers from nursing home facilities lead to large expenses for the facilities and Medicare.
• The Situation-Background-Appearance-Review and Notify (SBAR) form from the Interventions to Reduce Acute Care Transfers (INTERACT) program has been shown to help reduce hospital transfers

**What this paper adds**

• This paper evaluates the beneficial use of an APRN and the use of the SBAR form from the INTERACT program in three separate nursing home facilities.
• It provides insight into which hospital transfers from nursing homes can often be prevented by an APRN
• It provides findings that support the incorporation of an APRN in nursing homes to reduce the number of hospital transfers.

## 1 Introduction

### 1.1 Objectives

The focus of this project is to determine if the inclusion of an Advanced Practice Registered Nurse (APRN), as well as the Situation-Background-Appearance-Review-and-Notify (SBAR) form as part of the Interventions to Reduce Acute Care Transfers (INTERACT) program, will decrease the number of hospital transfers from three nursing home facilities. This will be determined by applying the following Purpose-Intervention-Comparison-Outcome-Timeframe (PICOT) question; For staff in three affiliated nursing home facilities (*P*), how does the implementation of a new treatment protocol that includes an evidenced-based assessment tool and APRN consults (*I*), compare to current care (*C*) impact timely APRN follow up and the number of hospital transfers (*O*) in eight weeks (*T*)?

### 1.2 Background

It has been found that more than 25% of nursing home residents have experienced a hospital transfer and that 47% of these transfers were labeled potentially avoidable (Ingber et al., 2017). Unnecessary or excessive hospital transfers may cause the patient to suffer increased mental, emotional, and physical distress (Brickman and Silvestri, 2019).

The healthcare system incurs a financial burden, with Medicare and Medicaid squandering an estimated two billion dollars a year on avoidable transfers (Ingber et al., 2017). Additionally, the nursing home facilities within the county this project was conducted receive a quality score based on multiple factors including rate of hospital transfers. The three facilities involved in this project lose funding from the county if a large percentage of their hospital transfers are deemed avoidable.

### 1.3 Framework

The conceptual framework applied in this project is the “Evidence-Based Practice Model for Staff Nurses,” created by Reavy and Tavernier (2008). This framework aims to engage staff nurses to apply evidence-based practice in their roles with a sense of purpose. As staff nurses become involved in these changes and outcomes, they hone their leadership abilities, critical thinking skills, and confidence. Beneficial outcomes are seen across the board when nurses change and adhere to new practices based on EBP. These outcomes include increased patient safety results, increased job satisfaction, decreased burnout, and the reduction of wasted resources and financial costs.

## 2 Methods

### 2.1 Specific aims

The proposed initiative mandated that an APRN, staffed by the county health department, was available to provide an in-person consult at each of the three nursing facilities to determine when a hospital transfer was necessary during two daytime shifts per week for the projects eight-week implementation period. The use of the SBAR screening tool found in the INTERACT Program, launched by The Center for Medicare and Medicaid Services (2020), was also utilized during this time period by the nursing staff at each of these facilities. The project lead provided information to the nursing staff which included training manuals, electronic delivery of recorded PowerPoint meetings, and examples of well-written and poorly-written SBAR forms. At weeks two, four, six, and eight, the project lead collected, electronically, the SBAR forms completed by the nursing staff with de-identified patient information. The project lead regularly reviewed this information and created additional recorded PowerPoints and outlines for electronic delivery to review questions, concerns, and address areas of excellence and areas in need of improvement.

### 2.2 Context

The physical and sociocultural makeup of the local environment included the county organization, the three facilities addressed in the PICOT question, and the hospital used for transfers. The county organization was dedicated to improving the quality of patient care while efficiently using resources and lowering overall costs. The structure within the organization included those who created and initiated / approved projects and programs, and those who participated hands-on with patient care. The organization was supportive of the project as it had been created several years prior but was unable to be implemented due to funding restrictions. Funding became available for this project through the county, with hopes that the results would help to increase further funding.

The nursing staff seemed resistant to the additional paper work included in this project despite understanding that a reduction in hospital transfers would ultimately lead to a reduction in paperwork. This barrier was addressed by hosting tele-format-based meetings during nursing shifts in which they could log on to ask questions and voice suggestions to the project lead as well as practice completing these forms while at work and avoid the use of their personal time.

### 2.3 Setting

The three nursing home facilities involved in this project were located within the same county and under the umbrella of that county's healthcare department, with any hospital transfers going to the same Emergency Department (ED). At each of these three nursing home facilities, the staff included Certified Nursing Assistants (CNA's), Licensed Practical Nurses (LPN's) and Registered Nurses (RN's). The average ratio for care was one RN per every six patients during a daytime shift and one RN per every eight patients during a nighttime shift. One Medical Doctor was on-call for these three nursing home facilities, as well as an additional three nursing home facilities within the county that were not included in this project.

### 2.4 Inclusion/exclusion criteria

The inclusion criteria were nursing home facilities within a 10 mile radius of each other that worked under the same county department and were affiliated with the same hospital. All nursing staff with the title of RN were included, regardless of their length of employment at that particular nursing home. All RN's hired during the eight-week project implementation timeframe were also included in the study. Exclusion criteria included any staff that did not hold the title of RN. The three nursing home facilities included in the project were selected among the six potential facilities as they had the highest rates of transfers consistently over the three quarters leading up to the project.

### 2.5 Intervention

An APRN was made available for two daytime shifts per week to perform in-person consultation and treatment when a hospital transfer was requested from any of the three nursing home facilities, assuming the call was not labeled as a time-sensitive emergency. Time-sensitive emergencies included patients exhibiting clear signs of heart failure, inability to breathe, and becoming cyanotic. When this occurred, the patient was immediately transferred to the ED. The APRN was available to all three nursing home facilities during the shift. When there was an overlap in calls, the first patient call was addressed while any following calls were automatically transferred to the ED. Upon arrival, the APRN provided treatment in-place or delegated this task to another staff member if that treatment was within their scope of practice. Examples of these treatments included, but were not limited to, the application of a nebulizing treatment, use of oxygen or changes to oxygen levels, the replacement of a foley catheter, prescribing of antibiotics, the infusion of a saline or dextrose solution by intravenous route, application of adhesive glue, bandages, or simple sutures, wound care and neurological exams, as well as speaking with the patient or the patient's family to alleviate anxiety. If the APRN believed the decision to treat in-place or transfer the patient was unclear, they were instructed to contact the Medical Doctor on-call. If the Medical Doctor on-call suggested a hospital transfer, then a hospital transfer occurred. Additionally, if the patient or the patient's designated medical power of attorney ultimately sought a hospital transfer after consultation by the APRN, regardless of the legitimacy of their request, they were transferred.

The second feature of the project design included incorporation of the SBAR form found in the INTERACT program. Pre-intervention, during each shift at any of these three nursing homes, the lead RN determined when to call for a hospital transfer based on personal opinion, experience, and comfort level. The project incorporated the use of the SBAR tool to provide consistency to the request for hospital transfers. The project lead led training and education sessions at the beginning of the project and consistently throughout the project. Due to constraints of COVID-19 these sessions were held on a HIPPA compliant tele-format through the county system. The staff learned how to use the tool, the tool's importance, and had an opportunity to practice using the tool and ask any questions they had. The project lead compiled information packets explaining the new protocols which were electronically distributed to the RN staff at each facility. Examples of well-written and poorly-written SBAR forms were provided and reviewed. Communication channels were kept open between the facilities staff members, the county health department organization, and the project lead to determine if adherence was occurring and if staff encountered any hurdles. Additional education and training sessions were conducted to support the staff and maintain compliance with the new protocols. The completed SBAR forms were collected bi-weekly.

The QI tool found in the INTERACT program was also used at each of the three nursing home facilities to determine how the staff felt towards using the SBAR form and integration of the APRN. These forms were collected bi-weekly with the completed SBAR forms.

### 2.6 Observations

The impact of the intervention was assessed by comparing rates of hospital transfers from these three selected nursing home facilities pre and post intervention, comprising a total of 16 weeks. These rates were compared per nursing home, and collectively. The county health department utilizes the data tracking program eMeds Elite by ImageTrend. The FirstWatch surveillance system is used in this data tracking program. This data included only de-identified patient information and was HIPPA compliant.

The evaluation plan developed was chosen so that a clear comparison could be made utilizing only objective interval data. This type of data lends itself to concise statistical analyses. The change in hospital transfer rates is discussed in the “Results” section of the paper.

Unfortunately, it was difficult to determine if the observed outcomes were solely due to the described interventions. The interventions had an immediate and direct impact on the reduction of hospital transfers, but also have the potential to create further change as the nursing staff become more acquainted with the SBAR forms and an APRN is hired on a full-time basis. While the interval data collected was unable to reveal if the decrease in hospital transfers was definitively from the applied interventions, the QI forms completed by the nursing staff helped to provide additional insight into the potential for continuation of the program.

### 2.7 Variables and data

Study variables included the number of hospital transfers from three nursing home facilities pre-intervention (control group) and post-intervention (intervention group). The independent variable included the application the SBAR form from the INTERACT program and the integration of an APRN to work with nursing home staff at three facilities, over eight weeks. The dependent variable was the number of hospital transfers that occured in response to these interventions. Data analysis was based on the objective interval data collected by the FirstWatch surveillance system. This data was collected every two weeks, with mid-point and end-point data collection reviews conducted at weeks four and eight to ensure accurate collection methods.

The use of the SBAR screening tool found in the INTERACT Program, launched by The Center for Medicare and Medicaid Services (2020), has been proven effective. This screening tool was used to aid the decision of hospital transfers. Ouslander et al. (2016) asserts that the SBAR and QI tools found within the INTERACT program met reliability and validity standards with a *P* value of 0.01, while Kane (2017) states that the SBAR form through the interact program revealed *p* < 0.01 with regards to reducing preventable hospital admissions from nursing home facilities. The INTERACT program tools are labeled free for public use.

The SBAR tool provided the nursing staff a guide to help aid their decisions regarding the necessity of a hospital transfer. The QI Tool, also part of the INTERACT program, helped to provide a prompt for reflection on the transfers that occurred to look for patterns and areas of improvement. The use of this QI tool was not part of the PICOT question, and so is not referenced in the “Results” section but helped to guide updates to the provided training information.

Compliance with the project interventions was monitored throughout the eight-week period by the project lead. This included confirming the use of an APRN by the county as well as the consistent use of the SBAR and QI forms by the nursing staff at the three nursing home facilities. These forms were collected on a bi-weekly basis to ensure that they were being completed in a correct and timely manner. Regular communication was maintained with the county health organization, the preceptor involved with this project, and the three nursing home facilities to ensure that all aspects of the project were being implemented to the extent originally proposed and agreed to. The IRB Office of Capella University reviewed this study and it was determined to not meet the federal regulations definition of Human Subjects Research. Therefore, IRB oversight was not needed.

## 3 Results

### 3.1 Findings

Originally, the PICOT question proposed a 10% reduction in overall hospital transfers at the end of the eight-week period. The reductions in hospital transfers seen are as follows: Facility one decreased an average of 59 hospital transfers to 38 hospital transfers when comparing the eight weeks prior to intervention and post-intervention. The percentage decrease was 35.6%. Facility two decreased from 17 hospital transfers to 6 hospital transfers when comparing the eight weeks prior to intervention and post-intervention. The percentage decrease was 64.7%. Facility three decreased from 39 hospital transfers to 31 hospital transfers when comparing the eight weeks prior to intervention and post-intervention. The percentage decrease was 20.5%. When combined, the three facilities decreased from 115 hospital transfers to 75 hospital transfers when comparing the eight weeks prior to intervention and post-intervention. The percentage decrease was 34.8%. This level of reduction in the number of hospital transfers that occurred alongside the intervention was much higher than originally anticipated. There was no missing data.

### 3.2 Impact

The nursing home facilities that responded quickly and enthusiastically to the project completed thorough training sessions, encouraged their nursing staff to attend optional meetings on the importance of the project, and encouraged them to take the time to complete detailed SBAR and QI forms.

A distinct barrier to project implementation at one of the facilities was engaging the nursing staff while adhering to the IRB restrictions set in place due to COVID-19. With the support of the county health organization, the project lead was able to work with the nursing home facilities to conduct Q&A sessions with the nursing staff via tele-format while the nurses were on their shift. This allowed the nurses to avoid using any personal time engaging with the project.

### 3.3 Analysis

The data collected consisted of the number of hospital transfers from the three nursing home facilities. The data was collected through the data tracking program eMeds Elite by ImageTrend; the program used by the county health department. The FirstWatch surveillance system is used in this data tracking program. The collected data was stored in this FirstWatch surveillance system with project lead granted secure access. The information from the SBAR and QI tools was also collected and reviewed, with both forms meeting reliability and validity standards with a *P*-value of 0.01 (Ouslander et al., 2016).

The data produced by the surveillance system is considered ordinal, while the data gleaned from the SBAR forms is considered nominal.

The statistical methods used included the mean difference between the pre-intervention period average and the post-intervention period average. The SPSS software was utilized for data analysis. These statistical tests produced clear results indicating a decrease in hospital transfers by these three facilities.

### 3.4 Interpretation

The use of an APRN to reduce hospital transfers was beneficial, with the APRN being able to prevent a hospital transfer in 45.5% of the cases they saw. The use of the SBAR forms allowed the nursing staff the opportunity to detail the reasoning for the transfer request which helped to aid a final transfer decision, often revealing that one was unnecessary.

In this project, the county chose to conduct follow-up reports on each patient that had been treated in place by the APRN 48–72 h after they were seen. No patients seen and treated by the APRN during this project experienced any adverse health events, nor were they dispatched to the hospital subsequently, according to these follow-up calls.

Originally, there was a concern that the unfolding of the COVID-19 pandemic would limit the findings due to stricter parameters regarding training sessions and data collection than were initially anticipated. However, ultimately, the COVID-19 pandemic may have increased the nursing home facilities eagerness to participate in the program in an effort to keep nursing home patients out of the ED.

The costs of this program are easily justified by the cost savings associated with the initiative. Costs of the program include the SBAR and QI tools along with their delivery to the nursing staff through training sessions. The SBAR and QI tools are accessible and free for distribution, with the contingency that they not be re-sold for profit. This leaves the only costs associated with this aspect to be the physical printing of the forms and the nurses hourly wage while attending training sessions as decided by the nursing home facility. The largest foreseeable cost of the intervention is the salary to maintain an APRN on staff. This cost will vary based on the regional pay for APRN's as well as the amount of shifts the APRN is on duty. The county involved with this project hired an APRN for two daytime shifts per week with plans to increase these shifts to include weekends and nights if additional funding is obtained.

## 4 Discussion

### 4.1 Limitations and assumptions

Initial assumptions included that the nursing home facilities would maintain consistent staffing ratios as well as a consistent population base. It was also assumed that data would be collected consistently despite the hurdles and ever-changing conditions of the COVID-19 pandemic.

Limitations begin with the short duration of the project. While the facilities were aware that the project would be implemented on a specific start date after IRB approval had been obtained, they were not provided the training materials until the start date. The inclusion of only three facilities could also be considered a limitation whereas the inclusion of more facilities within the county or state could have produced more insight. An additional factor that may have limited internal validity of the project results was the level of involvement and commitment each facility had to the project. One of the three facilities was extremely responsive while one of the facilities was delayed and disorganized possibly leading to skewed results.

Lastly, the final limitations were those connected to the COVID-19 pandemic. This project was originally created before the COVID-19 virus emerged and included plans for the project lead to make regular site visits to each of the three facilities. These site visits would have included meetings in which the nursing staff would have been encouraged to discuss their experiences and feelings regarding the new implementations. Unfortunately, due to COVID-19 regulations and IRB approval contingencies, no in-person training or data collection was permitted.

Several efforts were taken to minimize these limitations. While project implementation and data collection could not officially start until IRB approval was granted, the nursing home facilities were made aware that a project including the incorporation of an APRN and SBAR forms would be occurring in the near future so that they could alert their nursing staff. While the IRB contingencies based on COVID-19 prevented in-person staff training and data collection, meetings were held via a secure tele-format. The nursing home facilities and nursing staff members were provided the project lead's direct contact information along with pre-scheduled meeting times. The nursing staff were given permission to attend these tele-meetings while at work and were not required to use any personal time. Additionally, the project lead had coffee and pastries delivered to the three facilities at weeks four and eight on the day they were scheduled to submit their completed SBAR and QI forms as a sign of appreciation for their involvement.

### 4.2 Sustainability

This project is sustainable on a large scale as it does not require an overhaul of most nursing home facilities current procedures. Most nursing staff members are already acquainted with some form of the SBAR form making integration of the SBAR and QI forms from the INTERACT program achievable. APRN's are also becoming more integrated in healthcare, with RN's specifically understanding the APRN role as it is an extension of the RN role. As mentioned previously, the cost of a preventable hospital transfer can result in over a thousand dollars of lost revenue, which is easily countered by the cost of training materials and the addition of an APRN on staff, making these interventions an appealing way for nursing homes to increase patient care and staff satisfaction while decreasing costs.

After reviewing the successful results of this project, the county health organization where this project was completed has chosen to continue the use of the EBP implemented in this project for these three facilities. They plan to increase the number of nursing home facilities involved to cover a larger portion of the county, as well as consider bringing rehabilitation facilities into the fold. They plan to increase the number of shifts with an APRN available, and expand to night and weekend shifts.

### 4.3 Recommendations

Recommendations for further research focus on obtaining larger quantities of data, which could help to determine the efficacy of the interventions. The inclusion of more facilities over a longer period could better determine if the results seen were from the interventions or other factors. Additionally, an APRN was only hired for two daytime shifts per week. If an APRN had been available for a larger amount or variety of shifts the results may have been different. As reported by the county health organization, the APRN was able to intervene and prevent five transfers out of the eleven they participated in, resulting in a 45.5% decrease. All training materials and forms were provided solely in the English language. While the county in which the project was implemented requires nursing staff to pass an English proficiency test, this may not be the case in some areas, and some nursing staff may have felt more comfortable completing the forms had they been presented in their native language.

### 4.4 Future practice

Recommendations for future practice include creating a clear goal for the involved facilities, and receiving consistent feedback from the nursing staff regarding the SBAR and QI forms. This can include staff appreciation meetings and/or events, as well as emphasizing that a decrease in hospital transfers will ultimately lead to less paperwork.

## 5 Conclusion

This project has shown that the use of the SBAR tool alongside the incorporation of an APRN into nursing home facility protocols can reduce the number of hospital transfers that occur. With a reduction in hospital transfers comes a reduction in patient injuries during transfer, transfer-induced bouts of anxiety or delirium amongst patients, and financial costs to the facilities, the hospitals, and the county. The project is easily sustainable as the cost of educating nursing staff on these tools and hiring an APRN for several shifts a week is quickly offset by the savings seen as each hospital transfer can cost the county thousands of dollars. This project can spread to other facilities and districts, each determining changes that would best benefit their needs.

## Funding sources

None.

## Declaration of Competing Interest

None.
